# Prognostic values of pretreatment neutrophil-to-lymphocyte and platelet-to-lymphocyte ratios in endometrial cancer: a systematic review and meta-analysis

**DOI:** 10.1007/s00404-019-05372-w

**Published:** 2019-11-25

**Authors:** Liwei Ni, Jialong Tao, Jianhao Xu, Xuya Yuan, Yuming Long, Na Yu, Runhong Wu, Yusong Zhang

**Affiliations:** Department of Oncology, The Second Affiliated Hospital of Soochow University, Suzhou, 215004 Jiangsu People’s Republic of China

**Keywords:** Endometrial cancer, Neutrophil-to-lymphocyte ratio, Platelet-to-lymphocyte ratio, Prognosis

## Abstract

**Purpose:**

Elevated inflammatory markers, including neutrophil-to-lymphocyte ratio (NLR) and platelet-to-lymphocyte ratio (PLR), have been identified as poor predictors of survival in several malignancies. This meta-analysis was performed to quantify the effect of pretreatment NLR and PLR on the survival of patients with endometrial cancer (EC).

**Methods:**

This review systematically searched for relevant publications in databases of PubMed, Embase, and the Cochrane Library. Pooled hazard ratios (pHRs) with 95% confidence intervals (95% CIs) were determined and used to explore the association between inflammatory markers and overall survival (OS) and disease-free survival (DFS) in a random-effects model. Subgroup analysis, sensitivity analysis, and publication bias were also conducted in this meta-analysis.

**Results:**

Nine articles comprising 3390 patients were included. NLR higher than the cutoff was associated with a shorter OS (pHR = 2.22, 95% CI 1.77–2.78) and poorer PFS (pHR = 1.81, 95% CI 1.35–2.41). Patients with elevated PLR had high risk of decreased OS (pHR = 1.99, 95% CI = 1.51–2.61) and unfavorable PFS (pHR = 2.02, 95% CI 1.45–2.80).

**Conclusions:**

Elevated NLR and PLR during pretreatment are biomarkers of poor prognosis in patients with EC.

**Electronic supplementary material:**

The online version of this article (10.1007/s00404-019-05372-w) contains supplementary material, which is available to authorized users.

## Introduction

Endometrial cancer (EC) is the most common malignancy of the female reproductive system in developed countries [1]. The age-adjusted incidence and mortality rates of EC rose steadily in period 1978–2013 [2]. 5-year survival accounts for 74–91% of women in the early stages [International Federation of Gynecology and Obstetrics (FIGO) stages I or II]. However, for patients diagnosed with stage III or IV disease, 5-year survival rate decreased to 57–66% and 20–26%, respectively [3]. Therefore, identifying reliable and feasible biomarkers is needed for the early detection of patients with EC, development of individualized treatments, and implementation of follow-up protocols.

Survival of patients with EC depends on prognostic factors, such as age at diagnosis, comorbidities, tumor diameter, positive lymph nodes, histological grade and subtype, tumor grade, lymphovascular space involvement (LVSI), and FIGO stage [4]. Recently, studies have begun exploring prognostic values of inflammatory markers, including the neutrophil-to-lymphocyte ratio (NLR) and platelet-to-lymphocyte ratio (PLR), as biomarkers of systemic inflammatory responses associated with cancer development and progression [5–7]. NLR and PLR are useful prognostic indicators in different solid tumor types, such as head and neck squamous cell carcinoma [8], nonsmall cell lung cancer (NSCLC) [9], and breast cancer [10]. However, the relationship between NLR, PLR, and survival in EC remains obscure. Jiang et al. [11] demonstrated that a higher level of PLR was not significantly associated with overall survival (OS) in EC in a combined analysis of three studies. Ding et al. [12] identified that PLR and NLR greater than the cutoff was associated with poorer OS. Comert et al. [13] found that PLR was an independent prognostic marker for OS, but NLR was not a significant indicator. Prognostic values of NLR and PLR in EC are unclear. Hence, we conducted this meta-analysis to determine the predictive effect of pretreatment NLR and PLR on the OS and disease-free survival (DFS) of women with EC.

## Methods

### Search strategy

A comprehensive literature search was carried out for potentially eligible studies. We searched the PubMed, EMBASE, and Cochrane databases systematically using the following terms “platelet lymphocyte ratio” (OR “neutrophil lymphocyte ratio” OR “NLR” OR “PLR”) AND “endometrial cancer” (OR “endometrial carcinoma”) AND “prognosis” (OR “overall survival” OR “disease-free survival” OR “OS”, OR “DFS”). The search was updated in March 2019. We also manually screened the observational studies in the reference lists to identify other relevant publications. This study was performed following the Preferred Reporting Items for Systematic Reviews and Meta-analyses (PRISMA) guidelines [14].

### Selection criteria

Eligible studies must fulfill all of the following criteria: (1) the full text must be searchable in English; (2) the study population was histopathologically diagnosed with EC; (3) all patients with EC underwent complete blood count (CBC) prior to treatment; (4) the cut-off values of pretreatment hematologic parameters (NLR or PLR) were obtainable; and (5) hazard ratios (HRs) and their 95% confidence intervals (CIs) were reported on the association between pretreatment NLR and PLR and prognostic outcomes (DFS or OS). Duplicate articles, conference abstracts, reviews, letters, editorials, case reports, and laboratory studies were eliminated. Two authors (Liwei Ni and Jialong Tao) screened the candidate publications independently and reached a consensus after cross-checking. Cohen’s kappa statistic was used to assess inter-rater agreement (SPSS version 24. 0, SPSS Inc, Chicago, IL, USA).

### Data extraction and quality assessment

Two authors (Liwei Ni and Jialong Tao) independently extracted information from the selected studies. These information included the following variables: last name of first author, publication year, country, study design, duration, follow-up period, sample size, histological type, FIGO stage, tumor grade, treatment methods, interval time between a CBC blood test and treatments, cut-off values of NLR or PLR, and HRs with corresponding 95% CIs for OS and DFS. HRs with 95% CI in multivariable analyses were preferred if available. The Newcastle–Ottawa Scale (NOS, scores of 0–9 stars) was used to evaluate the quality of the included studies, and articles with NOS scores ≥ 6 were regarded as high-quality studies. Two reviewers assessed each study independently and reached a consensus after discussion.

### Statistical analysis

The primary endpoints of the selected studies were survival outcomes, including OS and DFS. The pooled HRs (pHRs) with 95% CIs were calculated to assess the prognostic values of NLR and PLR on EC. The Chi-square test and *I*^2^ statistic were used to evaluate the statistical heterogeneity among studies. *P* < 0.10 and *I*^2^ > 50% indicated significant heterogeneity, and the random-effects model was applied to calculate the pHR. Moreover, we conducted a sensitivity analysis to detect the source of heterogeneity. At least 10 studies were required to check for the existence of publication bias by constructing a funnel plot [19]. Thus, publication bias was examined using the Egger’s test. A two-tailed *P* value less than 0.05 was considered statistically significant. All data analyses were performed using Stata 14.0 (Stata Corporation, College Station, TX, USA) and SPSS 22.0 (SPSS Inc, Chicago, IL, USA).

## Results

### Literature search

**Fig. 1:**
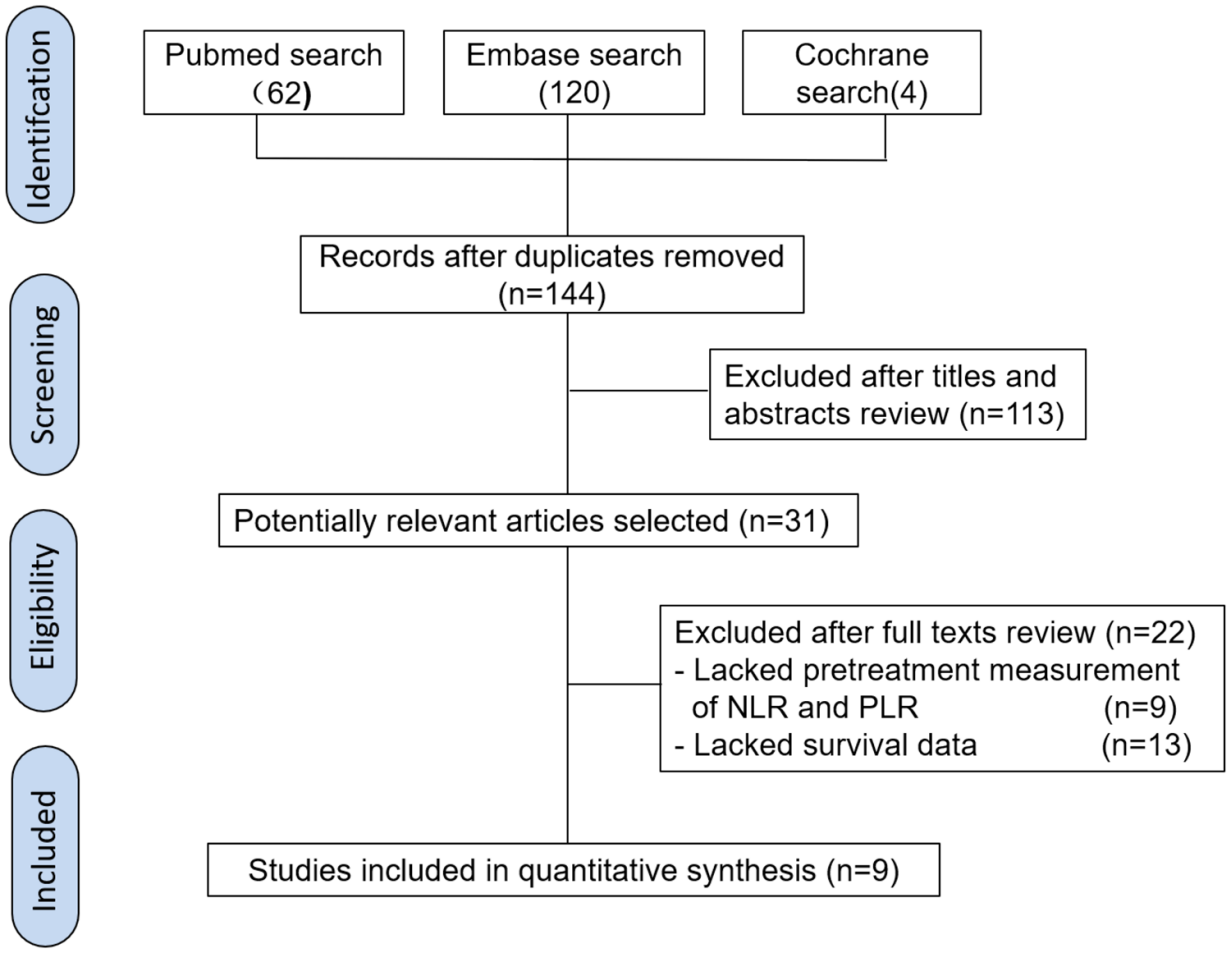
Literature search process

The flow diagram illustrates the literature selection process (Fig. 1). Initially, 186 articles were identified by searching three databases. Then, 42 duplicate records were found and removed. After reviewing titles and abstracts, the remaining 31 studies were further screened for eligibility. Among these remaining studies, 13 were excluded, owing to lack of survival outcome data, and 9 articles lacked of NLR and PLR data. Ultimately, nine retrospective cohort studies were involved in our meta-analysis. The kappa statistic indicated a high degree of consistency in study selection between two reviewers (*κ* = 0.95).

### Study characteristics and quality assessment

The characteristics and quality evaluation results of the included studies are depicted in Table 1. Nine cohort studies consisted of 3390 participants with EC. All of these studies reported survival outcomes for NLR, and seven of them reported survival outcomes for PLR. NLR and PLR values could be measured before cancer therapy (surgery, chemotherapy, and radiotherapy). The median cut-off values for NLR and PLR were 2.405 (1.81–4.68) and 190.78 (168–250), respectively. In addition, the NOS scores of all eligible studies were ≥ 6, thus indicating high quality.

**Table 1:** Characteristics of included studies for meta-analysis

Author, year	Study design	Duration	Follow-up (months)	Number	Age (years)	Tumor stage (FIGO)	Histological type	Tumor grade	Treatment	Interval	Prognostic factor (cut-off values) and survival data (HRs and 95% CIs)		Adjusted covariates	NOS scores
Aoyama, 2019, Japan	Retrospective cohort study	2007–2013	125	197	Median, 59	I–IV	Endometrioid, other	1–3	S	NR	NLR (2.18) OS: 2.23 (0.68–9.96) in Ua;	PLR (206) OS: 2.28 (0.74–7.72) in Ma	Age at diagnosis, BMI, FIGO stage, histological type, malignant ascites	7
Cömert, 2018, Turkey [13]	Retrospective cohort study	2006–2016	Median, 24	497	Mean, 58	I–IV	Endometrioid, clear cell, serous, mucinous, mixed, undifferentiated	1–3	C, S, R	8 ± 6 days	NLR (2.06) DFS: 1.10 (0.56–2.15) in Ua; OS: 1.70 (0.66–4.40) in Ua	PLR (168) DFS: 1.17 (0.55–2.51) in Ua; OS: 2.91 (1.15–7.36) in Ma;	Stage	7
Takahashi, 2015, Japan [19]	Retrospective cohort study	2000–2010	60	508	Mean, 58	I–IV	Endometrioid, non-endometrioid	1–3	C, S, R	NR	NLR (3) OS: 2.47 (1.45–4.24) in Ua	NR	NR	6
Li, 2015, China [21]	Retrospective cohort study	2007–2009	Mean, 51.2	282	Median, 53	I–IV	Endometrioid, non-endometrioid	1–3	C, S	Within 2 weeks	NLR(4.68) OS: 2.298 (0.679–7.781) in Ma	PLR (250) OS: 0.993 (0.294–3.357) in Ma	Serum concentrations of CRP, D–D, CA125 and CA153	7
Haruma, 2015, Japan [17]	Retrospective cohort study	2002–2012	130	320	Median, 57.5	I–IV	Endometrioid, serous, carcinosarcoma, clear cell, mixed, adenosquamous, undifferentiated	1–3	C, S	Within a month	NLR (2.41) DFS: 1.693 (0.888–3.229) in Ma; OS: 3.318 (1.154–9.538) in Ma	PLR (175.72) DFS: 1.599 (0.922–2.772) in Ua; OS: 0.546 (0.192–1.552) in Ma	FIGO stage, tumor histology, myometrial invasion, cervical invasion, lymph node metastasis, ovarian metastasis, peritoneal cytology, serum CA125	8
Eo, 2016, Korea [16]	Retrospective cohort study	2005–2014	Median, 51.3	255	Median, 44	I–IV	Endometrioid, serous, mixed, clear cell, mucinous, squamous, undifferentiated	1–3	S	Within 2 weeks	NLR (2.4) DFS: 3.68 (1.55–8.76) in Ua; OS: 3.47 (1.20–10.05) in Ua	PLR (190.78) DFS: 3.08 (1.30–7.32) in Ua; OS: 2.89 (1.00–8.38) in Ua	NR	6
Ding, 2017, China [12]	Retrospective cohort study	2007–2013	Mean, 65.84	185	Mean, 59.29	I–IV	Type I, type II	1–3	C, S, R	Within 7 days	NLR (1.81) DFS: 2.71 (1.26–5.82) in Ma; OS: 3.91 (1.58–9.81) in Ma	PLR (186.4) DFS: 2.98 (1.66–5.34) in Ua; OS: 3.68 (1.76–7.69) in Ua	Tumor histology, FIGO stage	8
Cummings, 2015, UK [15]	Retrospective cohort study	2005–2007	Median, 81.5	605	Median, 65	I–IV	Endometrioid, serous, carcinosarcoma clear cell, mixed	1–3	C, S, R	Within 2 weeks	NLR (2.4) OS: 1.82 (1.27–2.62) in Ma	PLR (240) OS: 1.89 (1.30–2.75) in Ma	Age, FIGO stage, grade, histopathological subtype, LVSI	8
Matsuo, 2015, USA [18]	Retrospective cohort study	2003–2013	Median, 35	541	Mean, 52.1	I–IV	Endometrioid, serous, clear cell, others	1–3	S	NR	NLR (3) DFS: 1.65 (1.02–2.65) in Ua; OS: 2.18 (1.21–3.93) in Ua	NR	NR	6

### Correlation between NLR and EC survival

**Fig. 2:**
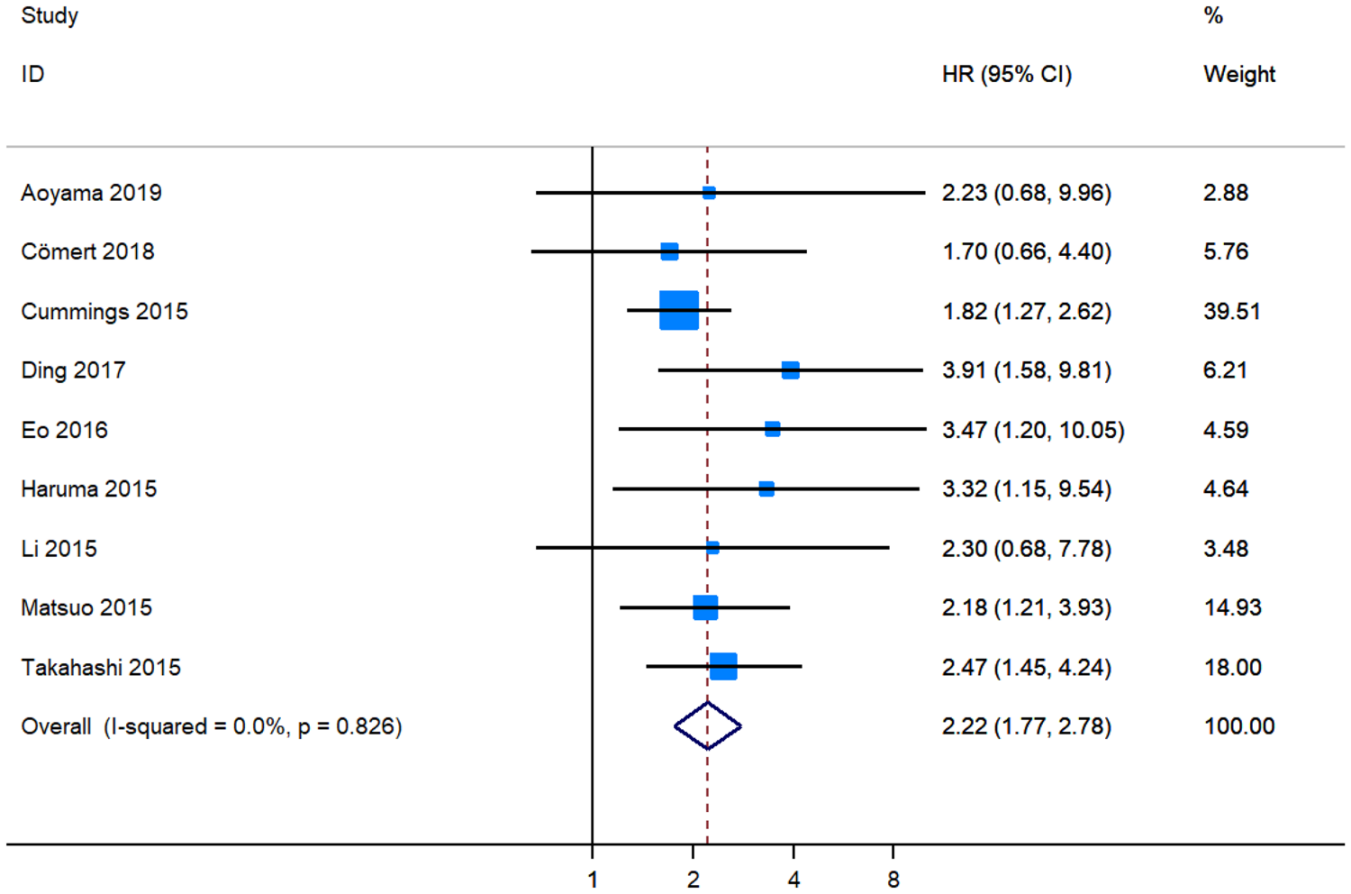
Meta-analysis of impact of NLR on overall survival of patients with endometrial cancer

Six original papers reported that NLR was an independent predictor for a shortened OS in patients with EC [12, 15–19], while NLR was not identified as a prognostic factor for OS in three studies [13, 20, 21]. The combined analysis of nine publications showed that the NLR values higher than the cut-off value predicted a worse OS (pHR = 2.22, 95% CI 1.77–2.78, Fig. 2) in individuals with EC [12, 13, 15–21].

**Fig. 3:**
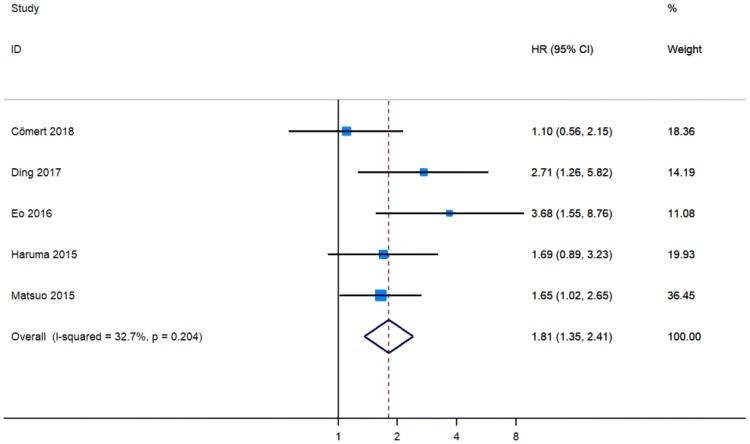
Meta-analysis of impact of NLR on disease-free survival of patients with endometrial cancer

Three cohort studies showed that NLR was an independent indicator of poor DFS in patients with EC [12, 16, 18], whereas two publications detected no significant relationship between NLR and DFS [15, 17]. Pooled analysis of five studies that included 1798 participants revealed that a higher NLR level was associated with worse DFS (pHR = 1.81, 95% CI 1.35–2.41, Fig. 3) [12, 13, 16–18].

### Correlation between PLR and EC survival

**Fig. 4:**
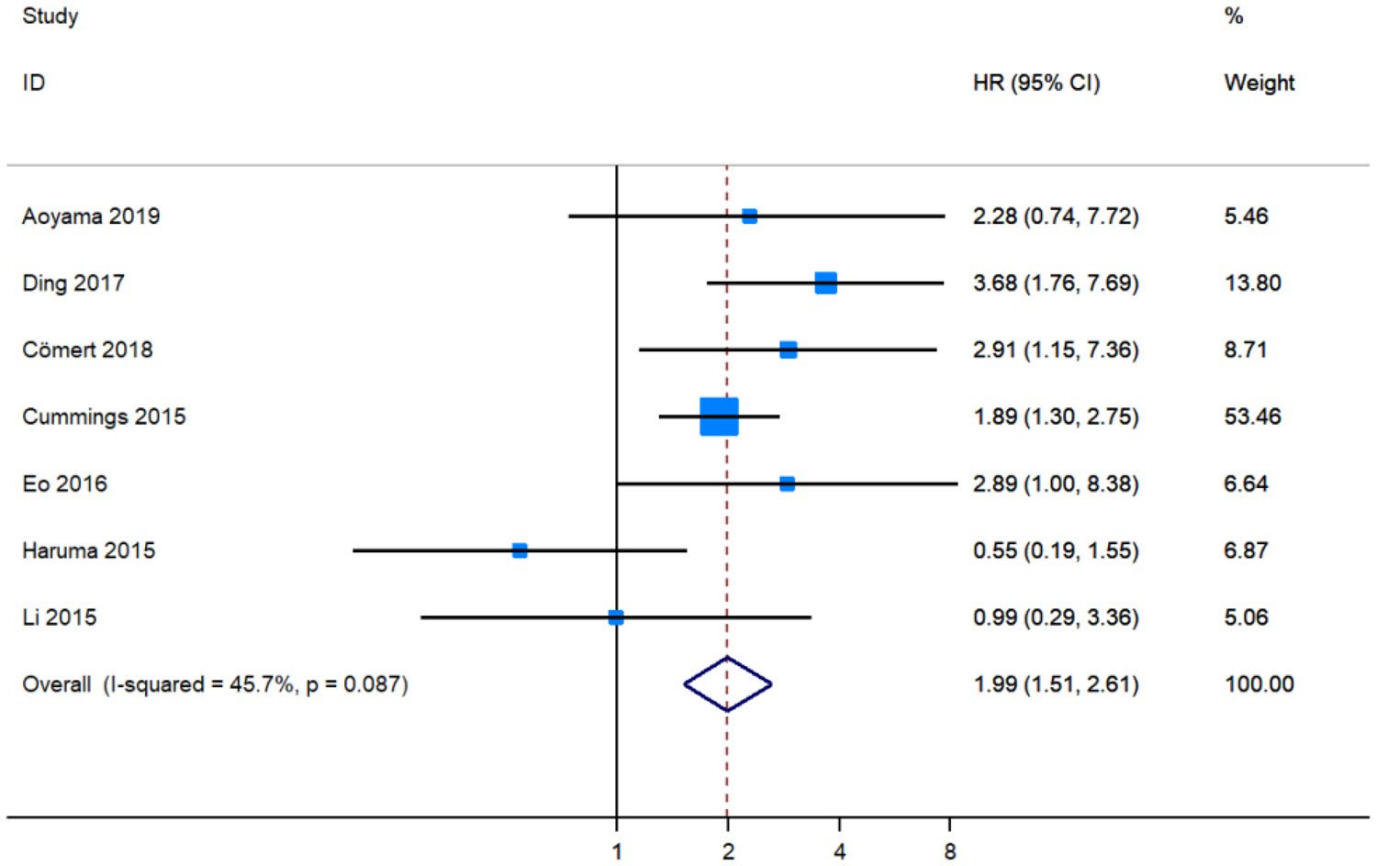
Meta-analysis of impact of PLR on overall survival of patients with endometrial cancer

Four original publications revealed that a higher level of PLR predicted a shorter OS in patients with EC [12, 13, 15, 16], while PLR was not considered as a prognostic marker for OS in three studies [17, 20, 21]. These seven articles comprising 2341 individuals with EC provided data on the relationship between PLR and OS [12, 13, 15–17, 20, 21]. The pooled results showed that patients with a higher PLR level had worse OS (pHR = 1.99, 95% CI 1.51–2.61, Fig. 4).

**Fig. 5:**
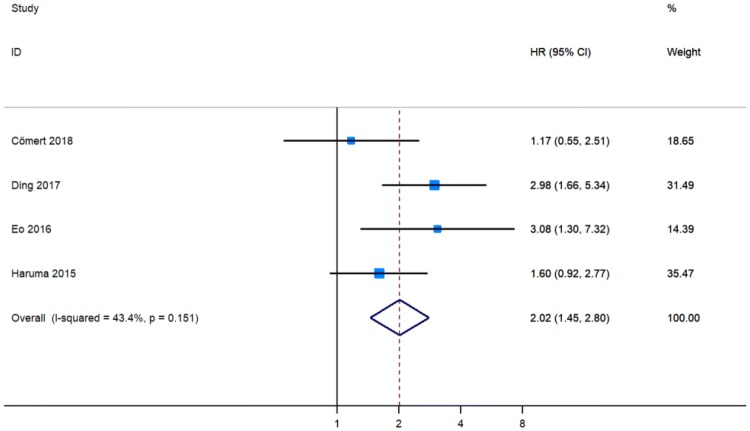
Meta-analysis of impact of PLR on disease-free survival of patients with endometrial cancer

Two studies showed that PLR higher than the cutoff indicated poorer DFS in patients with EC [12, 16], while two publications detected no significant association between PLR and DFS [13, 17]. These four studies involving 1257 patients calculated the pHR of PLR for DFS [12, 13, 16, 17]. Consequently, a higher level of PLR indicated a worse DFS (pHR = 2.02, 95% CI 1.45–2.80, Fig. 5).

### Heterogeneity and subgroup analysis

**Fig. 6:**
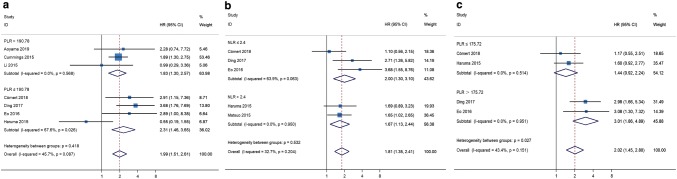
Meta-analyses of the association between PLR and overall survival (**a**), NLR and disease-free survival (**b**), PLR and disease-free survival (**c**) stratified by the median cut-off value among patients with endometrial cancer

**Fig. 7:**
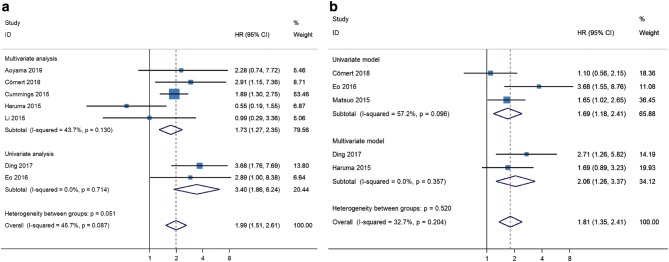
Meta-analyses of the association between PLR and overall survival (**a**) and NLR and disease-free survival (**b**) stratified by analysis method among patients with endometrial cancer

Because no significant heterogeneity was found between studies, the fixed-effects model was applied for meta-analysis of HR data. Considering the substantial heterogeneity observed in the pHRs of OS for PLR (*I*^2^ = 45.7%, *P* = 0.087, Fig. 4), of DFS for PLR (*I*^2^ = 43.4%, *P* = 0.151, Fig. 5), and of DFS for NLR (*I*^2^ = 32.7%, *P* = 0.204, Fig. 3), we conducted subgroup analyses to identify the source of heterogeneity. When stratified on basis of the median cut-off values, the pHRs of OS for PLR ≤ 190.78 (pHR = 2.31, 95% CI 1.46–3.65) and for PLR > 190.78 (pHR = 1.83, 95% CI 1.30–2.57) were similar to the combined estimate of subgroups (Fig. 6a). The results of heterogeneity analysis were unstable, with *I*^2^ for PLR > 190.78 decreasing to 0.0% and *I*^2^ for PLR ≤ 190.78 increasing to 67.6% (Fig. S1). In subgroup analysis of studies of NLR for DFS, no significant difference was found between NLR ≤ 2.4 group and NLR > 2.4 group (*P* = 0.532, Fig. 6b). The pHRs of DFS for PLR ≤ 175.72 and for PLR > 175.72 were 1.44 (95% CI 0.92–2.24) and 3.01 (95% CI 1.86–4.89), respectively (Fig. 6c). *I*^2^ decreased from 43.4 to 0.0% and heterogeneity between subgroups was significant (*P* = 0.027, Fig. 6c), indicating that the median cut-off value contributed to heterogeneity in the pHR of DFS for PLR. Based on the analysis method, the pHRs of OS for PLR in univariate analysis (pHR = 3.40, 95% CI 1.86–6.24) and in multivariate analysis (pHR = 1.73, 95% CI 1.27–2.35) were analogous to the overall estimate (Fig. 7a). *I*^2^ decreased to 0.0% in univariate analysis and it slightly decreased to 43.7% in multivariate analysis (Fig. 7a). The results revealed that analysis method was likely to be a source of heterogeneity among studies on the association between PLR and OS. However, the results of subgroup analysis showed that analysis method did not significantly contribute to heterogeneity in the pHR of DFS for NLR (*I*^2^ = 0.0% in multivariate analysis and *I*^2^ = 57.2% in univariate analysis, Fig. 7b).

### Sensitivity analysis and publication bias

The stability of our results in the random-effects model was measured using the trim-and-fill method. The pooled estimates showed no remarkable changes between the previous and new pHRs (Fig. S1, Online Resource). In addition, the new results did not significantly flip regardless of which publication was excluded (Fig. S2, Online Resource). None of the involved studies significantly disturbed the stability of the pooled estimate according to the corresponding sensitivity analysis. Furthermore, the potential publication bias was explored using the Egger’s test. We found no apparent publication bias among the cohort studies for NLR (*P* = 0.089 for OS and *P* = 0.311 for DFS) and PLR (*P* = 0.809 for OS and *P* = 0.954 for DFS).

## Discussion

A high level of NLR (or PLR) indicates poor cancer survival, according to the meta-analyses of various malignancies, including head and neck [8], lung [9], breast [10], renal [22], prostate [23], esophageal [24], pancreatic [25], colorectal [26], and hepatocellular cancers [27]. Conversely, a few combined analyses have revealed that NLR is not significantly associated with survival in patients with gastrointestinal stromal tumor [28], and PLR is not a reliable prognostic marker in patients with gastric cancer [29]. A meta-analysis by Jiang et al. [11] demonstrated that PLR is not significantly associated with OS in EC, but remarkably statistical heterogeneity shown in the combined analysis of three studies makes the pooled result unstable. In this present study, we conducted a similar meta-analysis and confirmed the predictive value of pretreatment NLR and PLR for the prognosis in women with EC. The pooled results provided evidence that elevated NLR and PLR indicated unfavorable survival outcomes in patients with EC.

Systemic inflammatory response is a basic feature of malignancy. Potential biomarkers of systemic inflammation, including C-reactive protein, modified Glasgow prognostic score, NLR, PLR, and lymphocyte-to-monocyte ratio, correlated with cancer survival [30]. Specifically, NLR is the most evaluated. Park et al. [31] found that an elevated NLR is associated with a poorer lymphocyte-mediated cytotoxicity against tumors measured by a lower density of tumor-infiltrating lymphocytes (CD3^+^ and CD8^+^ T cells) in individuals with colorectal cancer. Further studies for other malignancies are needed to identify the relationships between the systemic inflammatory response and local infiltration of T-effector cells. Intratumoral neutrophils may correlate with adverse survival outcomes in localized clear-cell renal-cell carcinoma [32]. In hepatocellular carcinoma, high neutrophil levels in peritumoral stroma are associated with cancer progression, indicating unfavorable survival [33]. Moreover, a meta-analysis revealed that a high density of intratumoral neutrophils predicts poor survival outcomes in patients with various solid tumors [34]. Han et al. [35] demonstrated a relationship between elevated NLR level in the peripheral blood and increased tumor neutrophil infiltration/decreased CD3^+^ T-cell infiltration. However, numerous studies should be conducted to further confirm the relationship between pretreatment NLR and immune-cell infiltration in diverse cancers and to clarify the mechanism on how NLR acts as a predictor of prognosis. PLR as another predictor is associated with cancer prognosis. Platelet count may increase secondary to the release of cytokines by tumor cells, thus stimulating megakaryocytes to produce platelets. Different types of cell factors, such as vascular endothelial growth factor (VEGF), epidermal growth factor (EGF), and interleukin-1β (IL-1β), are released during platelet activation and promote tumor growth and angiogenesis [36]. Platelets are involved in protecting tumor cells from cytolysis within the bloodstream, thereby contributing to hematogenous metastasis [37]. Zhang et al. [38] identified that tumor-infiltrating platelets are an independent indicator of adverse postsurgical prognosis in patients with pancreatic ductal adenocarcinoma. Nevertheless, the correlation among elevated platelet count, PLR, and tumor-infiltrating platelets is unclear.

Apart from the identification of prognostic values of the systemic inflammatory markers in patients with traditional cancer therapies (e.g., surgery, chemotherapy, and radiotherapy), the predictive effect of elevated NLR on adverse prognosis has been demonstrated in patients treated with immunotherapy, such as those with advanced NSCLC treated with nivolumab [39], those with metastatic renal-cell carcinoma treated with nivolumab [40], and those with advanced melanoma treated with ipilumumab [41]. Overall, the systemic inflammatory markers, namely, NLR and PLR, can be potentially clinically applied for predicting cancer prognosis. The NLR and PLR in the peripheral blood are easy to measure, cost effective, and noninvasive.

Our meta-analysis has limitations. First, all of the included studies were retrospectively observational studies; thus, difference in their unadjusted factors might lead to bias. Second, data of HRs in multivariable analyses were used if available, but a potential source of bias was found among HRs in two types of analysis methods. Nonetheless, the pooled results remained stable regardless of which publication for NLR or PLR was omitted. Third, the variable cut-off values of NLR (or PLR) might bring about noticeable heterogeneity, and the insight into whether these values were influenced by other conditions, such as pathogenic infections, remains uncertain. Furthermore, this combined analysis only involved nine studies comprising 3390 patients, and the sample size might not be large enough to support the outcome stability and to conduct detailed subgroup analyses. Although the contribution of the median cut-off value to heterogeneity in the pHR of DFS for PLR was statistically significant, the results of other subgroup analyses based on the median cut-off value or analysis method were unstable. Therefore, some noticeable heterogeneity and considerable bias may have existed among the studies.

## Conclusion

This meta-analysis demonstrated that high levels of pretreatment NLR and PLR as systemic inflammatory markers are associated with decreased OS and DFS in patients with EC. NLR and PLR are valuable prognostic biomarkers in most solid tumors, but their value in guiding treatment management needs further research. Moreover, the prognostic values of these systemic inflammatory biomarkers need to be further confirmed in prospective clinical trials for various malignancies. Further studies are also needed to explore the driving and regulatory mechanisms of cancer-related systemic inflammatory response and to search for potential therapeutic targets for cancer population.

## Electronic supplementary material

Below is the link to the electronic supplementary material.

Supplementary material 1 (PDF 236 kb)
