# Supplementary material for: Prognostic values of pretreatment neutrophil-to-lymphocyte and platelet-to-lymphocyte ratios in endometrial cancer: a systematic review and meta-analysis
Source: Arch Gynecol Obstet. 2019 Nov 25;301(1):251–61. doi: 10.1007/s00404-019-05372-w (PMC7028808; doi:10.1007/s00404-019-05372-w)
Supplement: Supplementary file 1 — Supplementary material 1 (PDF 236 kb) [file 404_2019_5372_MOESM1_ESM.pdf]

Journal name: Archives of Gynecology and Obstetrics

Article title: Prognostic values of pretreatment neutrophil-to-lymphocyte and platelet-to-lymphocyte ratios in endometrial cancer: a systematic review and meta-analysis

Author names: Liwei Ni*, Jialong Tao*, Jianhao Xu, Xuya Yuan, Yuming Long, Na Yu, Runhong Wu,

Yusong Zhang#

Affiliation: Department of Oncology, The Second Affiliated Hospital of Soochow University, Suzhou,

Jiangsu 215004, P.R. China

Corresponding Authors

#Yusong Zhang. e-mail: zhangyusong19@163.com.

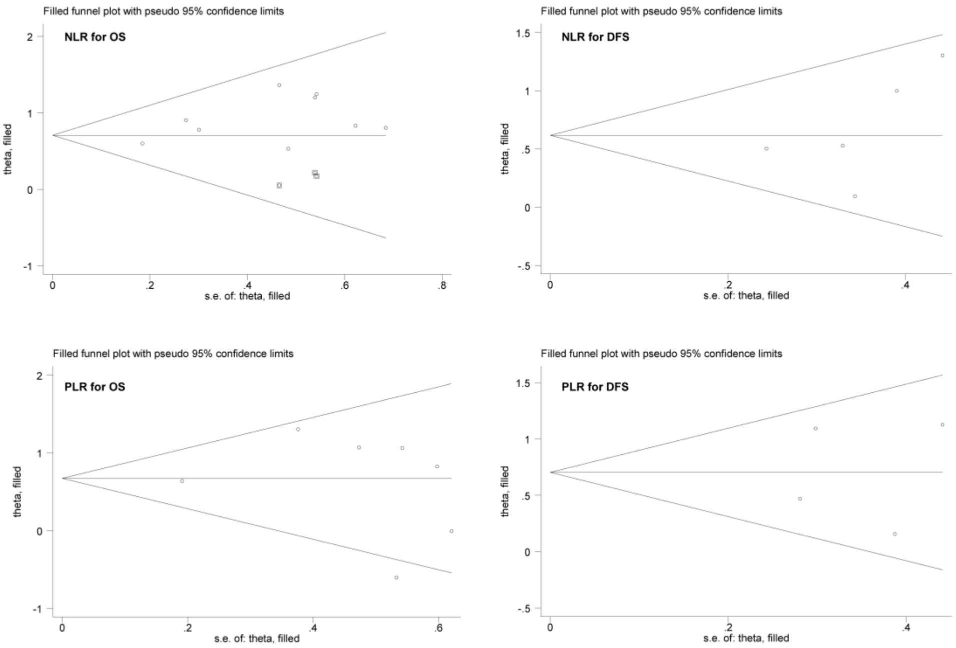

Fig. S1 Metatrim test of studies included in meta-analysis of NLR for OS (new pHR = 2.029, 95%CI = 1.642−2.508), NLR for DFS (new pHR = 1.805, 95%CI = 1.353−2.408), PLR for OS (new pHR = 1.987, 95%CI = 1.511−2.613), and PLR for DFS (new pHR = 2.017, 95%CI = 1.453- 2.799)

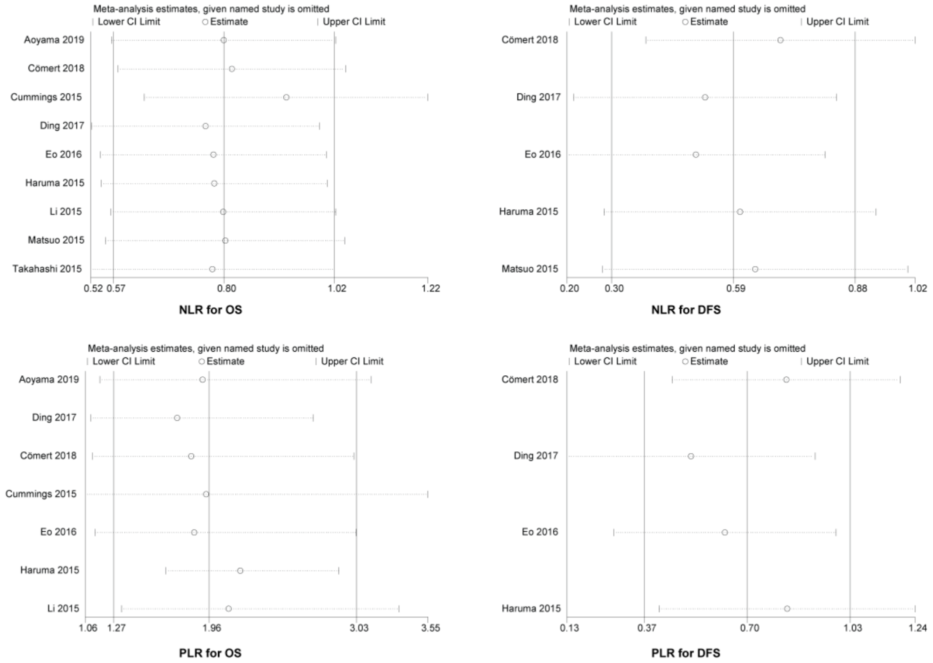

Fig. S2 Metaninf test of studies included in meta-analysis of NLR for OS, NLR for DFS, PLR for OS, and NLR for DFS
